# Thermal ablation in pancreatic cancer: A scoping review of clinical studies

**DOI:** 10.3389/fonc.2022.1066990

**Published:** 2022-11-29

**Authors:** William Farmer, Gary Hannon, Shubhrima Ghosh, Adriele Prina-Mello

**Affiliations:** ^1^ Nanomedicine and Molecular Imaging Group, Trinity Translational Medicine Institute, Dublin, Ireland; ^2^ Laboratory of Biological Characterization of Advanced Materials (LBCAM), Trinity Translational Medicine Institute, Trinity College Dublin, Dublin, Ireland; ^3^ Advanced Materials and Bioengineering Research (AMBER) Centre, Centre for Research on Adaptive Nanostructures and Nanodevices (CRANN) Institute, Trinity College Dublin, Dublin, Ireland

**Keywords:** thermal ablation, pancreatic cancer, radiofrequency ablation, high-intensity focused ultrasound, microwave ablation, laser ablation

## Abstract

**Background:**

Pancreatic cancer is a deadly cancer with a 5-year survival rate less than 10%. Only 20% of patients are eligible to receive surgery at diagnosis. Hence, new therapies are needed to improve outcomes for non-surgical candidates. Thermal ablation techniques can offer a non-invasive alternative to surgery.

**Aim:**

The aim of this review is to map the literature for the use of thermal ablative techniques: Radiofrequency ablation (RFA), High-intensity focused ultrasound (HIFU), Microwave ablation (MWA), and Laser ablation (LA) in the management of patients with PC.

**Methods:**

A search strategy was applied to PUBMED and EMBASE using keywords concerning pancreatic cancer, radiofrequency ablation, ultrasound ablation, laser ablation, and microwave ablation. The studies that fit this inclusion criteria were summarized in table format and results reviewed for interpretation.

**Results:**

72 clinical studies were included. Most of the included studies related to RFA (n=35) and HIFU (n=27). The most common study design was retrospective (n=33). Only 3 randomized control trials (RCT) were included, all of which related to RFA. Safety outcomes were reported in 53 of the 72 studies, and survival outcomes were reported in 39. Statistically significant survival benefits were demonstrated in 11 studies.

**Conclusion:**

The evidence for the benefit of MWA and LA in PC patients is limited. RFA and HIFU are safe and feasible therapies to be used in PC patients. Further RCTs where thermal techniques are standardized and reported are necessary in the future to elucidate thermal ablation’s clinical utility, and before an evidence-based decision on its routine use in PC management can be considered.

## 1 Introduction

Pancreatic cancer (PC) is a deadly disease, which according to GLOBOCAN cancer statistics accounted for 2.6% of new cancer cases, and 4.7% of cancer deaths globally in 2020 (1). This makes it the 7^th^ leading cause of cancer death worldwide (1). Surgery is the main curative treatment option for PC, but according to the American Cancer Society, fewer than 20% of patients are candidates for surgery (2).

For those with locally advanced and metastatic disease, chemotherapy (CHT) regimens like FOLFIRINOX have been shown to be effective in prolonging survival (3, 4). However, survival is dismal at this stage irrespective of CHT regimen, with the use of FOLFIRINOX (OS: 11.1 months) (4) and Nab-Paclitaxel+Gemcitabine (OS: 8.5 months) (5) giving a modest survival advantage over Gemcitabine (OS: 6.8 months (P<0.001)) (4) (OS: 6.7 months(P<0.001)) (5).

Radiotherapy (RT) can be used as chemosensitization in many cancers, but it’s chemosensitization in PC remains controversial. In the phase III LAP07 trial, after 4 cycles of induction gemcitabine +/- erlotinib, patients with locally advanced pancreatic cancer (LAPC) whose tumors were controlled were randomized to receive either CHT or chemoradiotherapy (CRT) for a further 2 months. There was no significant difference in OS in the CHT group (16.5 months) compared to the CRT group (15.2 months) (6). If the evidence for the combination of RT and CHT is inconclusive, perhaps thermal techniques could provide an opportunity to improve patient outcomes.

Thermal ablation has been defined as the use of temperatures >50°C for >4 min, or >512 CEM43°C (7) and has already demonstrated efficacy for managing other solid malignancies such as colorectal cancer and prostate cancer (8, 9). Ablative temperatures can be generated by several modalities including: RFA, LA, MWA, and HIFU. The preclinical evidence for the use of these techniques in PC models has been the subject of a systematic review from 2018 by Saccomandi et al. (10). The question that remains is whether these techniques can be effective in clinical studies of patients with PC.

The objective of this scoping review is to map the literature that has been published from clinical studies in this space. This review will focus on RFA, HIFU, MWA and LA, and will aim to address issues surrounding:

1. The safety and efficacy of these methods.
2. Standardization of these methods.
3. The potential future directions of this field.

## 2 Methods

This literature review was undertaken according to the Preferred Reporting Items for Systematic reviews and Meta-Analyses extension for Scoping Reviews guidelines (11).

### 2.1 Eligibility criteria

Texts were included in this review if they described an ablative intervention in PC patients, were in English, and if the full text was available *via* open access. Papers were excluded if they described *in vitro* studies, *in vivo* studies, review papers, abstracts, posters, or letters. Journal pre-proofs were included where available.

### 2.2 Information sources

The search was conducted on EMBASE and PUBMED. This was supplemented by relevant studies that were cited in the studies from EMBASE and PUBMED.

### 2.3 Search

**Figure 1:**
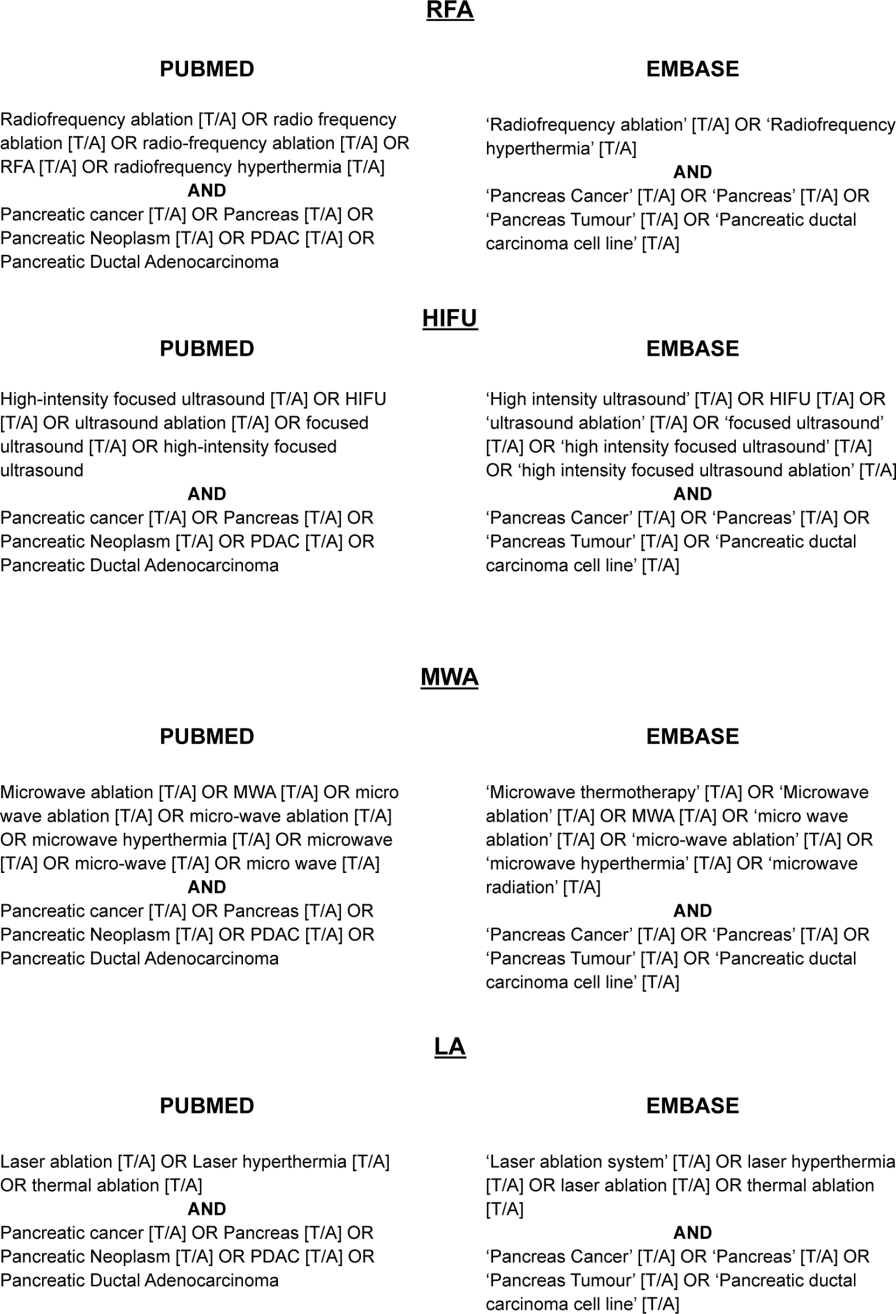
Search Strategies. These terms were applied to the title/abstract of texts in each database for each thermal technique.

Search strategies were undertaken for each modality on both databases. The strategies are described in detail in **Figure 1**.

### 2.4 Selection of sources of evidence

The abstracts of the results of each search were screened by the authors on the PUBMED and EMBASE databases. When the full text could not be retrieved automatically, the authors searched the internet using the article title. When abstracts, letters, and posters were retrieved by the automated feature, the authors searched the internet for corresponding full articles. The retrieved full texts were inspected, and if they fit the inclusion criteria, included in the review.

### 2.5 Data recorded

Data was sought for: year of publication, country of origin, aim of study, stage of disease, patient number, concurrent treatment course, size and type of lesion, parameters of the ablation procedure (frequency utilized/power transferred/energy generated/thermal dose), outcomes measured, results of the study, side effects reported, and the timing of the delivery of the ablation.

## 3 Results

**Figure 2:**
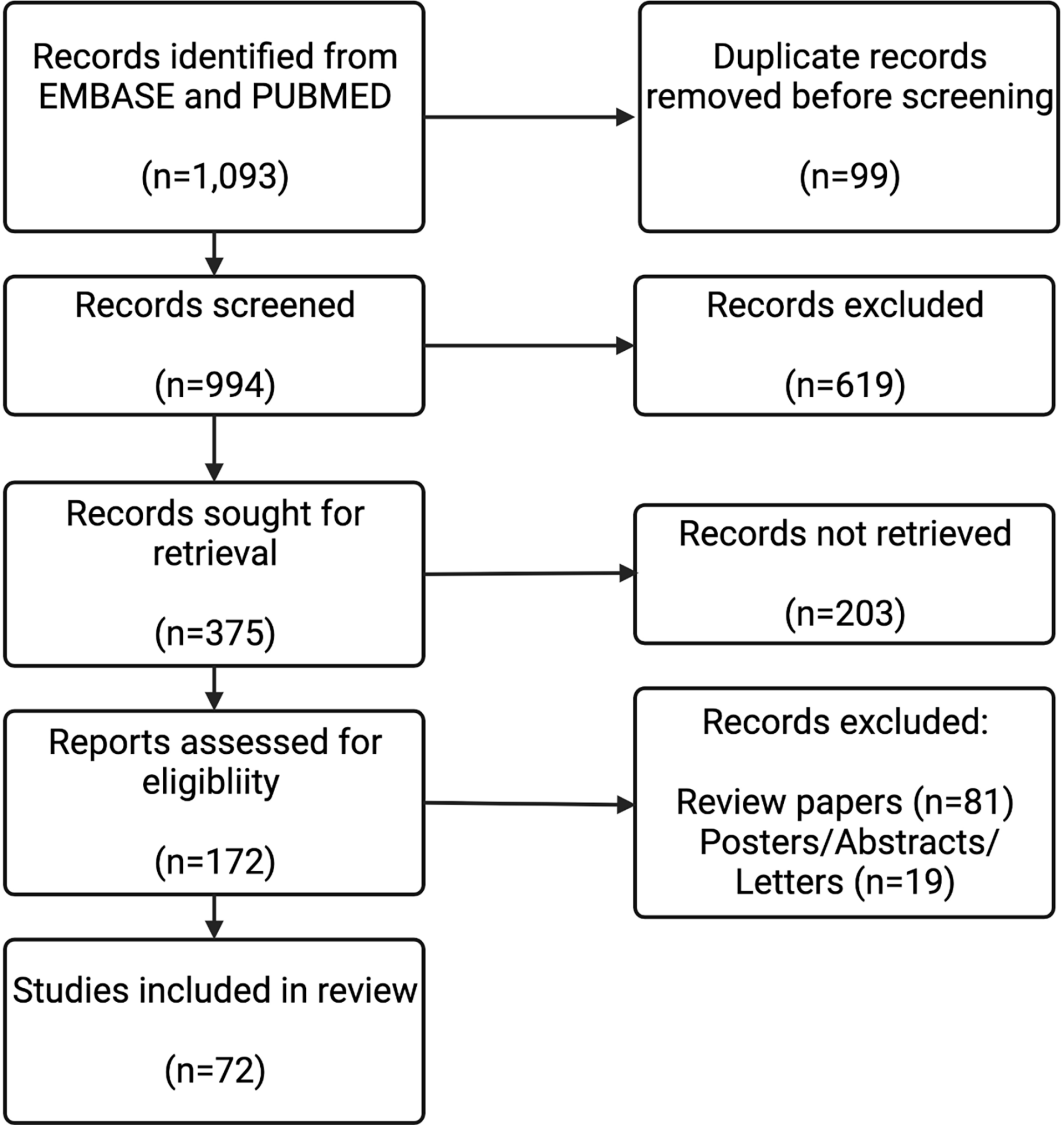
Study Selection Flow Diagram. The results from the 8 search strings described (n=1,093) were screened for duplicates. These results from each string were combined, and then screened by their abstracts (n=994). Full texts for the results that were deemed to be relevant from the abstract were sought (n=375). Full texts were assessed (n=172). Review papers (n=81), and posters/abstracts/letters (n=19) were excluded. 72 full-text records were included in this review.

The search strings resulted in 994 papers for screening. After screening and retrieval, 172 papers were assessed for eligibility. 100 of these were review papers, posters, abstracts, or letters, which left 72 papers to be included in this review. The flowchart summarizing the exclusion process is depicted in **Figure 2**.

**Figure 3:**
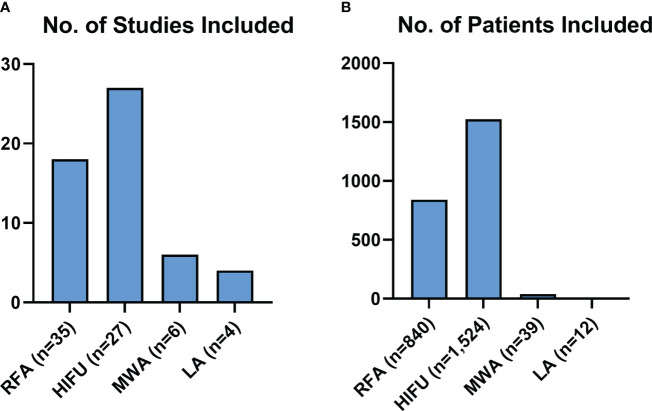
Review Population Graphs. Figure 3 describes the makeup of all the subjects of this review for each modality, both in terms of studies **(A)** and patients included **(B)**.

**Figure 3** represents the population included in this review, both in terms of studies for each modality, and numbers of patients receiving each modality. There were considerably more studies and patients detailing RFA and HIFU than MWA and LA.

### 3.1 Radiofrequency ablation

#### 3.1.1 Summary of results

The search strategy for RFA resulted in 35 clinical studies being included in this review, making it the most described ablation technique for PC. The studies detail the use of RFA in the treatment of pancreatic ductal adenocarcinoma (PDAC) (n=637), neuroendocrine tumors (NET) (n=10), and cystic lesions (n=8).

**Figure 4:**
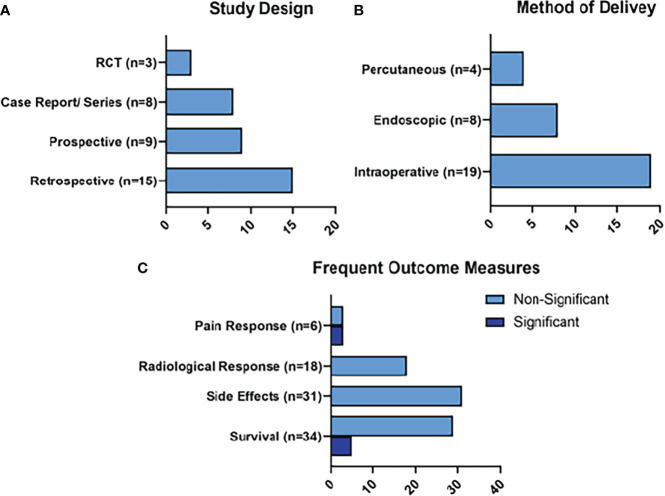
Summary of RFA Results. Figure 4 represents the main characteristics of the RFA studies included in this review in terms of study design **(A)**, method of delivery of RFA **(B)**, and frequent outcome measures **(C)**. Frequent outcome measures are subdivided into those that reached statistical significance, and those that did not.

The frequent outcomes measured in the studies detailing RFA treatment were survival measures (n=34), radiological responses (n=18), and pain responses (n=6), as represented in **Figure 4**. Statistically significant survival benefits were reported in 5 of the studies, while a significantly improved pain response was noted in 3 studies. Side effects were reported in 31 of the studies. The side effects of note reported for RFA were peri-pancreatic fluid collections (n=13; 1.54%), pancreatic fistula (n=11; 1.3%), venous thrombosis (n=10; 1.19%), pancreatitis (n=7; 0.83%), and gastrointestinal hemorrhage (n=5; 0.59%).

The target ablation temperature was reported in 14 studies, and it ranged from 30°C to 105°C. The power settings were reported in 15 studies. The power settings ranged from 5-10W (12) to 200W (13). The duration of the delivery of RFA was described in 26 of the studies, with the longest duration of ablation being 60 minutes (14), and the shortest being 50 seconds (15).

#### 3.1.2 Notable studies

Two RFA studies included in this review were concerned with immunomodulation/immunostimulation (16, 17). In 1986, Falk et al. added immunostimulatory compounds (Copovithane/PZ-73C/NED-137) to the treatment of a cohort of 77 PC patients receiving CHT and RFA treatment. They discovered significant percentage survival benefits at 6 months (60.1% vs. 29.8% P<0.008), and 12 months (35% vs. 6% P<0.001) in the patients receiving immunostimulation over those who were not (17).

More recently, Giardino et al. performed a prospective study of the immunomodulatory properties of RFA in the treatment of LAPC (16). Patients were excluded if they had any previous medical oncology treatment. RFA was applied intraoperatively with ultrasound (US)-guided RFA at 90°C using the Uniblate single cool-tip probe™, which has a built-in thermocouple for thermal monitoring. The mean application time was 6 minutes. 30% of patients experienced complications. These were 1 hemorrhage (managed conservatively), 1 ulcer, and 1 pancreatic pseudocyst (16). They analyzed two immunological parameters: serum cell populations (CD8 and CD4 T cells, Treg cells, NK cells, dendritic cells, and monocytes), and serum cytokines (IL-6, CCL-5, SDF1, VEGF, TGF-B, TNF-a). Both CD4 and CD8 T cells demonstrated a significant increase in number from day 3 to day 30 (16). There was a particular increase in effector memory T cells, while no expansion of Treg cells were observed. There was significant enhancement in dendritic cells at day 30 which are fundamental in presenting tumor-associated antigen (16).

The highest quality study assessing the utility of RFA as an up-front therapy comes from Frigerio et al. who published results from a RCT in 2021 (18). They compared the use of RFA with subsequent CHT or CRT (group A), against standard CHT or CRT only (group B). The only requirement for the CHT/CRT regimens used for both cohorts was that they had to have a documented efficacy for treating PDAC that was at least as good as gemcitabine-based therapy. The lack of restriction on the choice of CHT/CRT regimen was chosen because the authors did not want to preclude the participants from receiving novel therapies.

100 patients with LAPC were recruited for this study. 16 of the 48 patients randomized to group A did not receive RFA due to findings of metastases (n=10), or safety concerns (n=6). US-guided RFA was performed during a laparotomy using a Uniblate™ device with the temperature never exceeding 90°C. One month after RFA, group A received CHT or CRT (18).

The OS in group A was 14.2 months, while the median OS in group B was 18.1 months (p=0.639), demonstrating a non-statistically significant reduction in OS. The PFS in group A was increased to 8 months compared to 6 months in group B however this did not reach statistical significance (p=0.570). Three grade B pancreatic fistulas, one delayed gastric emptying, and one abdominal collection requiring treatment were observed as RFA-associated complications (18). Currently, this is the only published RCT on the effect of RFA on OS in LAPC. While the results are disappointing, when they are taken in the greater context of advancements made in LAPC treatment (novel CHT combination therapies) the results may not be very relevant to the current LAPC treatment landscape. This will be explained further in the discussion section.

### 3.2 High-intensity frequency ultrasound

#### 3.2.1 Summary of results

The search strategy yielded 27 studies that referred to the use of HIFU in patients with PC. 928 of the tumors treated were PDAC, there were 2 NET treated, and the remainder were referred to as PC or unspecified.

**Figure 5:**
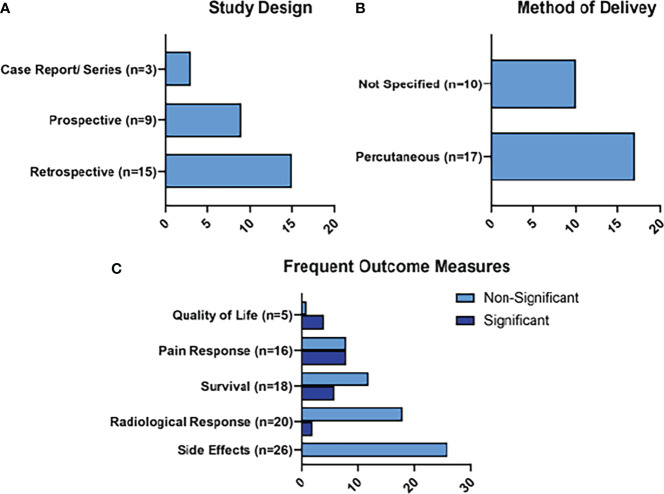
Summary of HIFU Results. Figure 5 represents the main characteristics of the HIFU studies included in this review in terms of study design **(A)**, method of delivery of HIFU **(B)**, and frequent outcome measures **(C)**. Frequent outcome measures are subdivided into those that reached statistical significance, and those that did not.

The most common outcomes were radiological responses (n=20), survival outcomes (n=18), pain responses (n=18), and quality of life responses (n=5), as represented in **Figure 5**. Two of the radiological responses, 6 of the survival responses, 8 of the survival responses, and 4 of the quality of life outcomes were statistically significant. Some of the more commonly reported side effects of HIFU treatment included: pancreatitis (n=9; 0.6%), pseudocyst formation (n=5; 0.32%), and skin burns (n=58; 3.8%). These skin burns were mild in 49 of the cases, and more severe in 9, sometimes requiring plastic surgery (n=3).

The power settings and/or energy administered was reported in 25 of the studies, and the timing of HIFU was reported in 23. The range of power settings was from 100 W (19) to 1,350W (20). The sonification times ranged from 725 seconds (21) to 6,000 seconds (19).

#### 3.2.2 Notable study

HIFU has been described as a surgical tool to facilitate resection. Wang et al. published a retrospective analysis of feasibility and safety of HIFU in 30 patients with BR disease (22). These patients had an *in-situ* gastric tube (which was removed during the subsequent operation) filled with degassed water to improve the acoustic path. The median power of the HIFU was 274 W ( ± 87 W) for an average sonification time of 1452 ( ± 370s). 7-9 days after HIFU, 27/30 patients underwent surgical resection. 18 of the patients who underwent surgery also had 21 days of gemcitabine regimen. From the original group of 30 patients, the total resectable rate was 90%. 25 of the cases were R0 resections, while 2 were R1 resections (22). This resection rate of patients with BR disease has been shown to vary across studies. A prospective study in 2019 including 249 BR patients determined the resectable rate to be 24.1% after neoadjuvant treatment (23), while a 2019 retrospective study of 151 reported their resectable rate as 63.6% (24). It appears HIFU can offer a potential effective alternative to neoadjuvant CHT for patients with BR PC.

HIFU has further been described as a therapy in combination with CHT. Li et al. retrospectively compared the use of HIFU in combination with the S-1 (Tefagur/Gimeracil/Oteracil) CHT regimen (n=61), versus S-1 regimen alone (n=59) in metastatic gemcitabine-refractory PDAC (25). S-1 was administered twice daily for one week. This was repeated every 3 weeks until disease progression or toxicity. 2-6 cycles (median= 4) were applied to each patient. Further details of the HIFU treatment were not reported other than the fact that it was delivered percutaneously under US guidance (25). Overall survival was 10.3 months for the HIFU and S-1 group compared to 6.6 months for S-1 monotherapy. PFS was 5.1 months compared to 2.3 months, respectively. In the combination group, 1 patient had a complete response, and 15 had partial response (RECIST 1.1). In the monotherapy group, 5 patients showed a partial response. There was a significant benefit in the proportions of responders in the HIFU group. No grade 3 or 4 adverse events were noted, while patients in the combination group experienced transient nausea, vomiting, anorexia, and diarrhea. Some slight skin burns also occurred in the HIFU group (25).

### 3.3 Microwave ablation

#### 3.3.1 Summary of results

There were less clinical studies available concerning the use of MWA than for RFA and HIFU. From the search strategy, 6 clinical studies of MWA fitted the inclusion criteria. 4 studies treated PDAC, 1 study treated an intraductal papillary mucinous neoplasm (IPMN), and 1 study treated an insulinoma.

**Figure 6:**
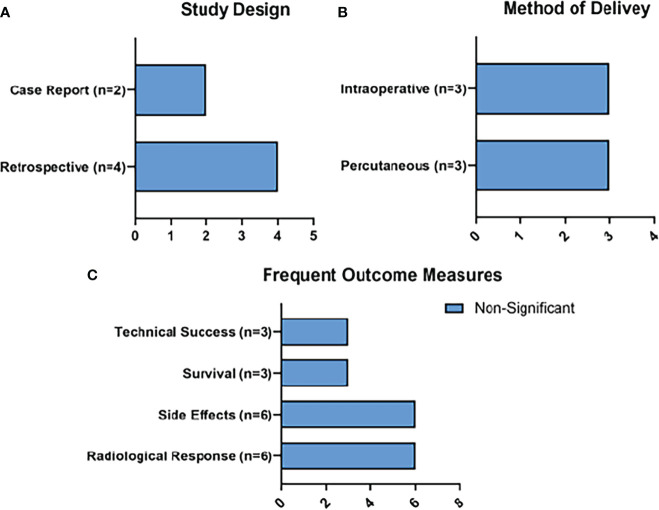
Summary of MWA Results. Figure 6 represents the main characteristics of the MWA studies included in this review in terms of study design **(A)**, method of delivery of MWA **(B)**, and frequent outcome measures **(C)**. None of the frequent outcome measures reached statistical significance.

Radiological responses were reported as an outcome measure in all 6 of the studies. Survival was reported in 3, and technical success was reported in 3 (**Figure 6**). Side effects were reported in all 6 of the studies. 3 liver or pancreatic abscesses were observed (7.7%), 2 pseudocysts were reported (5.12%), and 2 cases of severe local pain were reported (5.12%).

Temperature was not reported in any of the studies, while power was reported in two studies (20W and 100W) and mean cumulative energy output was reported in 1 (9,627W). Duration of the MWA was also only described in 3 studies, and averaged 148 seconds.

#### 3.3.2 Notable studies

MWA was notably utilized as a method of treating insulinomas. Egorov et al. detailed MWA performed on 7 patients (26). These patients had insulinomas and were symptomatic with hyperinsulinism at presentation. They were deemed unfit for surgery, or at high risk of postoperative complications. MWA was performed percutaneously, during a laparotomy, and laparoscopically. The treatment was effective in all patients to render them normoglycemic at 3 days, without any recurrence at the end of follow-up which was 31 months long (26). There were 2 pancreatic fistulas observed. 1 patient developed a pancreatic fistula 1 month after MWA which was drained (26).

### 3.4 Laser ablation

#### 3.4.1 Summary of results

From the search strategy, 4 clinical studies examining the use of LA fit the inclusion criteria. 11 patients were treated for PDAC, and 1 had an IPMN. Power settings in these studies ranged from 2W-5W, energy delivered ranged from 800J- 14,000J, and the study that detailed ablation time ranged from 200 seconds -600 seconds.

**Figure 7:**
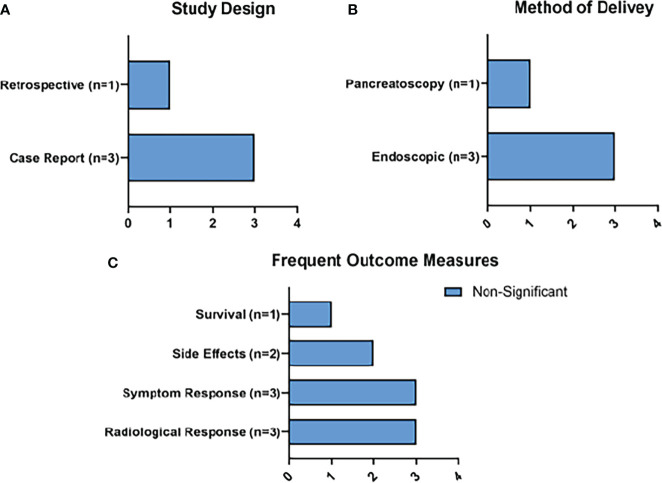
Summary of RFA Results. Figure 7 represents the main characteristics of the RFA studies included in this review in terms of study design **(A)**, method of delivery of MWA **(B)**, and frequent outcome measures **(C)**. None of the frequent outcome measures reached statistical significance.

Radiological response (n=3) and symptom response (n=3) were the most frequently reported outcomes. Survival outcomes were only reported in 1 study (**Figure 7**). Side effects were reported in 2 of the studies, with the only reported side effect of LA being 3 cases of peripancreatic fluid collections.

#### 3.4.2 Notable studies

In 2018, Di Matteo et al. conducted a small prospective cohort study of the feasibility and safety of endoscopic ultrasound-guided (EUS) LA in the treatment of locally advanced PDAC which was unresponsive to previous CRT (27). Feasibility was measured by CT imaging as evidence of coagulative necrosis post-ablation. Safety was measured by the occurrence of adverse events. They applied the LA at a different power (2-4W), energy (800-1,200J) and duration (200-600s) in each of the 9 participants to demonstrate safety and feasibility at a range of operating settings (27). CT scans at 24 hours, 7 days and 30 days after ablation demonstrated well-defined coagulative necrotic areas. The ablated areas decreased in all cases at 30 days. No major adverse events were recorded, but 3 patients showed peri-pancreatic fluid that spontaneously disappeared. Median overall survival was 7.4 months. It was determined that a power of 4 W and 1,000 J achieved the largest ablation volume without adverse effects, and concluded that EUS- guided LA was safe and feasible.

## 4 Discussion

The aim of this scoping review was to characterize the evidence on the use of RFA, HIFU, MWA and LA. The sources of evidence were heterogeneous, with the most common study design being retrospective review (n=33), followed by case studies (n=16). There were only 3 RCTs reviewed, and they were all for RFA (18, 28, 29). Based on the numerous studies detailing the use of RFA and HIFU compared to MWA and LA, it seems the interest of the scientific community is currently focused on RFA and HIFU. The interest in RFA in particular as a cancer therapy is further reflected in the report by Research Nester (a market research firm) that predicts the global gastrointestinal RFA systems market to grow at a compound annual growth rate of 6% from 2022-2030 (30). While many of the studies in this review demonstrated benefits to patients with the use of these techniques, the predominance of retrospective study design over RCTs is a serious limitation of the evidence for these techniques. Furthermore, there is a lack of standardization in the application of ablation in terms of temperature recorded, exposure time, and energy applied, which makes it difficult to compare results.

The evidence for the efficacy of these techniques depends on the outcome measured. Radiological tumor responses were commonly seen in these studies, and pain reduction was frequently reported in patients following treatment. Survival was reported in 56 of the studies, and 11 of these demonstrated statistically significant improvements in OS (12, 25, 31–33), median survival time (34, 35), and disease specific survival (33). However, the RCTs for RFA did not show this survival benefit (18, 29).

The occurrence of side effects and adverse events was reported in 53 of the 72 studies, and the majority were mild or moderate. The complications of note were pancreatitis (n=17), fistula formation (n=16), and pseudocyst formation (n=7). Skin burns occurred exclusively in HIFU studies (n=42). Grade I to Grade III burns accounted for 90% of the burns. In one of the HIFU studies (36), two of the burns were of grade III severity and required plastic surgery. The pancreatitis was generally classified as mild or moderate, except for in one HIFU study (36), and two RFA studies (37, 38) where it was severe.

### 4.1 Evidence for effect on overall survival

The goal of any cancer therapy is to prolong survival, and many of the trials address the effect that ablation has on OS. Most of the trials that have shown statistically significant improvements in OS take the form of retrospective studies, and while some of them have large patient numbers included, there has been a limited number of RCTs published that address the effect of ablation on OS.

A particularly notable retrospective study that showed a statistically significant improvement in OS is a study by Ning et al. (2019) that examined the outcomes of 523 cases of unresectable PDAC. 347 patients received HIFU treatment and gemcitabine, while 176 patients received gemcitabine monotherapy. OS was 7.4 months in the combination group compared to 6 months in the monotherapy group (P=0.004) (32). One of the main limitations of this study is a lack of randomization and potential selective bias. However, the improvement in OS is encouraging, and suggests that further studies could provide stronger evidence for the use of HIFU.

In a study that built on the retrospective evidence, Sofuni et al. (2021) performed a prospective clinical safety trial to evaluate the effects of HIFU for unresectable PC (34). 176 patients received HIFU and CHT, and 89 patients received CHT only. The CHT regimens in this trial included gemcitabine monotherapy, S-1 monotherapy, Gemcitabine plus S-1 therapy, Gemcitabine plus Nab-paclitaxel, and FOLFIRINOX. The median survival time after diagnosis was 21.3 months in the HIFU and CHT group compared to 9.5 months in the CHT only group (P<0.001) (34). Although this was a prospective study, there was a possible selection bias based on the timing of HIFU, prior therapy, and CHT regimen of the patients. A strength of this study, however, is that the included CHT regimens are more representative of current options for LAPC. Despite its lack of randomization, this study still adds to the evidence for ablation to prolong OS in LAPC and highlights the need for RCTs in the future.

The highest-quality RCT available on thermal ablation in PC to date was published by Frigerio et al. (2021) in which they randomized 100 patients with LAPC to receive either CHT/CRT from an oncologist, or up-front US-guided RFA followed by CHT/CRT (18). The only requirement for the CHT/CRT regimens used for both cohorts was that they had to have a documented efficacy for treating PDAC that was at least as good as gemcitabine-based therapy. The lack of restriction on the choice of CHT/CRT regimen was chosen in the trial design stage because the authors did not want to preclude the participants from receiving novel therapies. As we will see, this is part of the reason why the data from this RCT may have been less relevant at the date of publication.

This trial was conceived in 2013 following previous retrospective studies (37, 38) hoping to treat LAPC and achieve an OS that exceeded 14 months. The authors point towards two factors that they believe impacted their results, and that would have led them to consider another approach to this trial, in retrospect.

The first is that they did not recruit the desired number of patients to this trial. They estimated the required sample size to be 126 patients based on the primary endpoint of OS at 1 year. Only 100 patients were enrollled, and then there was a high dropout rate in the RFA arm of 33.3% due to findings of metastases or safety concerns (18). Ultimately, the study lacked power to detect any significant differences between groups.

The second issue was that in the prolonged period that it took to enrolll, treat, and follow-up on the patients, significant advancements in the treatment of LAPC were made. Namely, the acceptance and success of the combination therapies FOLFIRINOX and Nab-Paclitaxel and Gemcitabine. These advancements made the results of this trial less relevant to LAPC treatment at the time of publication, because many patients received ‘outdated’ CHT regimens. What the authors ultimately concluded from their work was that upfront RFA does not provide a benefit to LAPC patients and therefore shouldn’t be offered (18).

This is not to say, however, that RFA has no place in the management of LAPC. The PELICAN trial is currently in progress. This study is a multicenter superiority RCT examining the effect of second line RFA and CHT versus CHT only on OS in LAPC patients who have stable disease or partial response following at least 2 months of CHT therapy (39). Wherever possible, patients will receive FOLFIRINOX or nab-paclitaxel and gemcitabine. The primary endpoint will be OS, and secondary endpoints will include PFS, radiological tumor response, quality of life, pain, and immunomodulatory effects. The study is aiming to enroll 228 patients (40).

The PELICAN trial is designed to be more relevant to the current landscape of LAPC treatment than the RCT performed by Frigerio et al. When the first RCT was conceived, gemcitabine monotherapy was the dominant treatment for patients with LAPC, and so RFA was added as an upfront treatment. However, in the current era of FOLFIRINOX, improved OS and the possibility of downstaging and resection are known to be possible, meaning that LAPC patients should avail of these treatment regimens before trying less-established therapies. Therefore, this clinical trial of RFA as a second-line treatment should yield more representative evidence for the future application of RFA in the clinic.

### 4.2 Further potential applications of ablation

For patients with BR PC, RFA could be used as a surgical adjunct to improve resectability. Surgical resection is the only curative option for PC, but only 20% of patients are eligible at diagnosis (2). Furthermore, the rate of R0 resection (tumor-free margin of 1mm) can be low. In a cohort study conducted by Hank et al. in 455 patients who underwent upfront resection for PC, the R0 rate was 23.5%, the R1 (tumor free margin less than 1mm) resection rate was 22.9%, and the R2 (direct invasion of the margin) resection rate was 53.6% (36). They also showed that R0 resection rate was a significant prognostic factor for overall survival. The median OS was 62.4, 24.6, and 17.2 months for R0, R1 (>1mm), and R2 (direct) respectively (36).

In one notable study included in this review, Kumar et al. described the use of RFA as an adjunct to pancreaticoduodenectomy in 6 patients with locally advanced disease where blood vessel involvement prevented resection and vessel reconstruction (41). 4 of these 6 patients achieved R1 margin status after use of RFA, and there were no intraoperative complications. There could be potential for surgeons to use RFA in combination with resection to improve chances of R0 and R1 resections, and better patient outcomes.

The immunomodulatory effects of ablation could be a promising avenue of future clinical trials. The concept of combining RFA and immunostimulatory agents was actually first reported by Falk et al. in 1986 in patients with PDAC receiving RFA and CHT. Survival was significantly increased in patients with immunostimulation (17). More recently, Giardino et al. examined the effect that RFA has on the immune system. Following RFA performed after laparotomy, serum cytokines were not greatly modulated but a number of populations of immune cells were elevated (16). These findings should be treated with caution however, due to the small sample size and the possible confounding factor of post-surgery inflammation. If future larger clinical studies could replicate these findings however, there is rationale to combine thermal therapy with immunotherapies to ameliorate this largely immunosuppressive cancer.

### 4.3 Standardization

There are notable limitations to the studies included in this review. The standardization of how heat is delivered is not consistent across studies, which makes comparison difficult. Only 16 of the studies in this review included temperature readings (15 RFA and 1 HIFU). All of the RFA studies relied on thermal sensors incorporated into their RFA probes for this reading: Starburst XL, RITA Systems (n=5); Cool-tip, Radionics (n=3); Uniblate™, AngioDynamics (n=3); Celon POWER, Olympus (n=2); Habib™ 4X, AngioDynamics (n=1). The only HIFU study to report temperature readings was by Vidal-Jove et al. They reported that ‘the median intensity of treatment was 350W, which corresponded to a median temperature of 70°C (42). However, no detail is given about where and when this temperature reading was recorded.

The international working group on image-guided tumor ablation have published a standardization of terminology and reporting criteria (43). They say that temperature measurements should include precise specification of where the temperature was measured. Most of the studies that report temperature in this review provide this information, but two RFA studies do not include this detail, and the only HIFU study to report temperature doesn’t report this either (41, 42, 44). The standard reporting criteria also say that it should be specified when during the ablation the temperature measurements were acquired. This information is not clear in any of the studies included in this review. When the reporting of, and standardization of the ablation is inconsistent, it makes it very difficult to draw conclusions about their effects. For example, it could be that the statistically significant improvements in OS seen in the retrospective trials did not transfer to the RCTs due to inconsistent treatment deliveries.

To be able to report this information in clinical trial papers, accurate thermal monitoring must also be in place. Thermal probes can be positioned within the tumor volume and in healthy adjacent tissue to provide information about the temperature in the treatment volume, and to provide safety warnings at the desired ablation boundary. These probes can be used to monitor RFA treatment especially, however they are less suitable to monitor HIFU treatment because their placement negates HIFU’s main clinical advantage, in that it is non-invasive. For HIFU, MRI based thermometry can non-invasively monitor temperature in real time, but it is far more expensive. In the future, all studies should employ a recognized method of thermal monitoring, be consistent with consensus guidelines for tumor ablation (45), and the standardization of terminology and reporting criteria (43).

### 4.4 Future directions

The most probable future application of ablation will take the form of adjuvant second-line treatment in combination with CHT. The future progression of this therapeutic field clearly will rely on quality RCTs which detail standardized thermal dosages and delivery methods of thermal ablation according to international expert consensus. The last update was posted on the 1st of August 2017 which said that the trial was recruiting. As previously mentioned in the discussion section, the results of the PELICAN trial will also inform what direction this field goes in (40).

Another possibly interesting direction to pursue with ablation could come in the form of combination therapies with immunotherapies in order to turn an immunologically ‘cold tumor’ hot. In this vein, there is a phase II trial (NCT04156087) of patients with non-resectable PC to undergo minimally invasive MWA in combination with a CTLA-4 mAb, a PD-L1 mAb, and adjuvant gemcitabine. The study will examine PFS and is estimated to be completed in 2023. Should the data from this trial prove to be significant, the previously underwhelming response to immunotherapy seen in PC could be overcome, providing a new treatment option to the PC patients who require it most.

### 4.5 Conclusion

In conclusion, ablative techniques like RFA and HIFU still require more standardization and investigation before they can be applied confidently to PC in the clinical setting. The positive effects on OS that these techniques have demonstrated in numerous retrospective studies provides encouragement for the utility of these therapies. Ultimately however, progress will not be made until these benefits are translated to adequately powered RCTs that compare ablation techniques to current gold-standard treatment regimens for unresectable PCs. In order to achieve this and provide reproducible results across treatment centers and research groups, thermal monitoring and reporting of achieved temperatures in the tumor volumes must be standardized according to current consensus from international working groups. Beyond its effects on OS, these ablative techniques could have applications in combination with immunotherapies, as a surgical adjunct, or for palliation of PC-associated pain. However, it must be acknowledged that in some clinical scenarios, neoadjuvant thermal therapy is in competition with more established techniques like surgery and other tumor reducing strategies. All in all, thermal ablation remains an promising area of cancer research which merits further investigations.

## Author contributions

WF and GH contributed to conception and design of the review. WF performed the initial database search and wrote the original draft. SG and AP-M revised and contributed to the draft. All authors contributed to manuscript revision, read, and approved the final submitted version.

## Acknowledgments

We would like to acknowledge the partial funding provided by the TCD Translational Oncology Masters Programme and the EU H2020 Safe-N-Medtech project (Grant no. 814607).

## Conflict of interest

The authors declare that the research was conducted in the absence of any commercial or financial relationships that could be construed as a potential conflict of interest.

## Publisher’s note

All claims expressed in this article are solely those of the authors and do not necessarily represent those of their affiliated organizations, or those of the publisher, the editors and the reviewers. Any product that may be evaluated in this article, or claim that may be made by its manufacturer, is not guaranteed or endorsed by the publisher.

## Abbreviations

PC, Pancreatic cancer; RFA, Radiofrequency Ablation; HIFU, High-Intensity Focused Ultrasound; MWA, Microwave ablation; LA, Laser ablation; RCT, Randomized control trial; CHT, Chemotherapy; FOLFIRINOX, Folinic acid, fluorouracil, irinotecan hydrochloride, and oxaliplatin; MPC, Metastatic pancreatic cancer; OS, Overall survival; RT, Radiotherapy; LAPC, Locally advanced pancreatic cancer; CRT, Chemoradiotherapy; CEM43°C, Cumulative equivalent minutes at 43°C; PDAC, Pancreatic ductal adenocarcinoma; NET, Neuroendocrine tumor; US, Ultrasound; RECIST 1.1, Response evaluation criteria in solid tumors 1.1; CT, Computed tomography; BR, Borderline resectable.
